# Positive imagery cognitive bias modification (CBM) and internet-based cognitive behavioral therapy (iCBT): A randomized controlled trial

**DOI:** 10.1016/j.jad.2015.02.026

**Published:** 2015-06-01

**Authors:** Alishia D. Williams, Kathleen O’Moore, Simon E. Blackwell, Jessica Smith, Emily A. Holmes, Gavin Andrews

**Affiliations:** a School of Psychiatry, UNSW Medicine, University of New South Wales, Sydney, NSW, Australia; b Clinical Research Unit for Anxiety and Depression (CRUfAD), St. Vincent׳s Hospital, Sydney, NSW, Australia; c MRC Cognition and Brain Sciences Unit, Cambridge, UK; d Department of Clinical Neuroscience, Karolinska Institutet, Stockholm, Sweden

**Keywords:** Cognitive-bias modification (CBM), Internet-based therapy (iCBT), Major depressive disorder, Depression, Randomized controlled trial (RCT), Mental imagery

## Abstract

**Background:**

Accruing evidence suggests that positive imagery-based cognitive bias modification (CBM) could have potential as a standalone targeted intervention for depressive symptoms or as an adjunct to existing treatments. We sought to establish the benefit of this form of CBM when delivered prior to Internet cognitive behavioral therapy (iCBT) for depression

**Methods:**

A randomized controlled trial (RCT) of a 1-week Internet-delivered positive CBM vs. an active control condition for participants (*N*=75, 69% female, mean age=42) meeting diagnostic criteria for major depression; followed by a 10-week iCBT program for both groups.

**Results:**

Modified intent-to-treat marginal and mixed effect models demonstrated no significant difference between conditions following the CBM intervention or the iCBT program. In both conditions there were significant reductions (Cohen׳s *d* .57–1.58, 95% CI=.12–2.07) in primary measures of depression and interpretation bias (PHQ9, BDI-II, AST-D). Large effect size reductions (Cohen׳s *d* .81–1.32, 95% CI=.31–1.79) were observed for secondary measures of distress, disability, anxiety and repetitive negative thinking (K10, WHODAS, STAI, RTQ). Per protocol analyses conducted in the sample of participants who completed all seven sessions of CBM indicated between-group superiority of the positive over control group on depression symptoms (PHQ9, BDI-II) and psychological distress (K10) following CBM (Hedges *g* .55–.88, 95% CI=−.03–1.46) and following iCBT (PHQ9, K10). The majority (>70%) no longer met diagnostic criteria for depression at 3-month follow-up.

**Limitations:**

The control condition contained many active components and therefore may have represented a smaller ‘dose’ of the positive condition.

**Conclusions:**

Results provide preliminary support for the successful integration of imagery-based CBM into an existing Internet-based treatment for depression.

## 1 Introduction

Depression is a global health problem, estimated to become the leading cause of burden of disease worldwide by 2030 (World Health Organization, 2008). The limitations in efficacy and accessibility of current treatments for depression have led to increasing recognition of the need for treatment innovation, such as development of easier to access, and more cost-effective psychological therapies (Simon and Ludman, 2009). Cognitive science provides one promising avenue for such work (Holmes et al., 2014). Research has begun to demonstrate that simple emotion training computerized procedures, known as cognitive bias modification (CBM; Macleod, 2012) may be used to selectively target biases implicated the onset, maintenance, and recurrence of depression.

The CBM paradigm most often researched in depression focuses on two cognitive targets; mental imagery and interpretation. Participants repeatedly practice imagining positive resolutions for ambiguous situations, and through this imagery CBM aims to train a bias to automatically imagine positive resolutions for novel ambiguous situations encountered in daily life. Depressed individuals struggle to imagine positive future events (Holmes et al., 2008; Morina et al., 2011) and tend to interpret ambiguous information negatively rather than positively – a negative interpretation bias (Butler and Mathews, 1983). Training positive mental imagery (via repeated positive imagery generation) in conjunction with positive interpretation (via consistently positive resolutions of the ambiguous situations; cf. Mathews and Mackintosh, 2000) may therefore be particularly helpful in reducing symptoms of depression, via targeting these particular processes synergistically (Holmes et al., 2009a). Experimental studies have demonstrated that a single session of imagery CBM could increase positive affect and positive interpretation in healthy volunteers (e.g., Holmes et al., 2009b, 2006). Subsequent preliminary studies in clinical samples found reductions in symptoms of depression and negative interpretation following a one-week schedule of seven CBM sessions (Blackwell and Holmes, 2010; Lang et al., 2012; Torkan et al., 2014).

One possible therapeutic deployment of such a brief treatment module would be to combine CBM with existing evidence-based computerized treatments such as Internet cognitive behavioral therapy (iCBT; Andersson, 2009). While both CBM and iCBT aim to change negative cognitive biases, they do so via different methods: iCBT via explicit ‘top down’ cognitive evaluation and behavioral experiments, and CBM via a potentially more direct ‘bottom up’ cognitive training approach. While there are several ways in which these two approaches could be combined, one possibility is that positive imagery CBM may provide a useful preparatory treatment module prior to engaging in iCBT. For example, the trained interpretive bias may make it easier to generate alternative thoughts. Further, repeated practice in imagining positive outcomes for activities during CBM may facilitate anticipation of positive outcomes from behavioral tasks during iCBT. Such increased positive anticipation could potentially have more generalized benefits, such as increased interest in everyday activities more broadly, which may be beneficial in particular for reducing anhedonic symptoms of depression (Blackwell et al., 2015).

In the first trial to evaluate a combined positive imagery CBM plus iCBT intervention we (Williams et al., 2013b) randomized patients diagnosed with a major depressive episode to an 11-week intervention (CBM+iCBT) or a wait-list control (WLC). Results supported treatment superiority over WLC on all outcome measures (Hedges *g*=.74–.98, 95% CI .11–1.62). Results also demonstrated that the putative causal mechanism, change in interpretation bias, at least partially accounted for the reduction in depression symptoms following CBM. Further, 65% of patients who completed the combined intervention evidenced clinically significant change (reaching high end-state functioning).

This first trial provided encouraging results of the integration of two Internet-based technologies into an efficacious and acceptable form of treatment delivery for major depression. However, the comparison group was a wait-list control. In the current RCT we aimed to carry out a more rigorous test of CBM via comparison to a closely-matched active control condition (i.e., exposed to identical intervention procedures minus one or more of the hypothesized active treatment ingredients; cf. Clarke et al., 2014b). Following Lang et al. (2012), we used a control condition in which only one of the putative active ingredients was removed. Specifically, the control condition was identical to the positive imagery CBM, except that no valence-specific training contingency was established: only half of the ambiguous training scenarios were resolved positively, whereas the rest were resolved negatively. To test the additive benefit of positive imagery CBM when delivered in combination with iCBT, all patients completed an established iCBT program for depression following the 1-week CBM intervention.

### 1.1 Hypotheses

We predicted that patients randomized to positive imagery CBM (positive condition) would evidence significant reductions on primary measures of depressive symptoms (PHQ9, BDI-II) and interpretive bias (AST-D), and on a secondary measure of psychological distress (K10) following the 1-week CBM intervention. We predicted superiority of positive imagery CBM over CBM control (control condition) on these measures following the 1-week intervention phase. We also predicted significant reductions on measures of depression (PHQ9, BDI-II,) and psychological distress (K10), as well as disability (WHODAS), repetitive negative thinking (RTQ), and anxiety (STAI) following completion of iCBT. Although we anticipated comparable outcomes following completion of iCBT, as the first study to investigate the potential additive benefit of positive imagery CBM prior to iCBT (relative to an active comparator), we tested for between-group superiority of the positive condition on measures of depression and distress following the combined intervention. Following a recent RCT in which a four-week positive imagery CBM intervention significantly reduced anhedonic symptoms of depression compared to an active control (in the absence of between-group differences for symptoms of depression as a whole (Blackwell et al., 2015)), we carried out post-hoc analyses to investigate whether we could also find significant benefits for anhedonia. Finally, we predicted that the within-group benefits would be maintained at 3-month follow-up.

## 2 Methods

### 2.1 Participants and recruitment

**Fig. 1. Study Flowchart.**
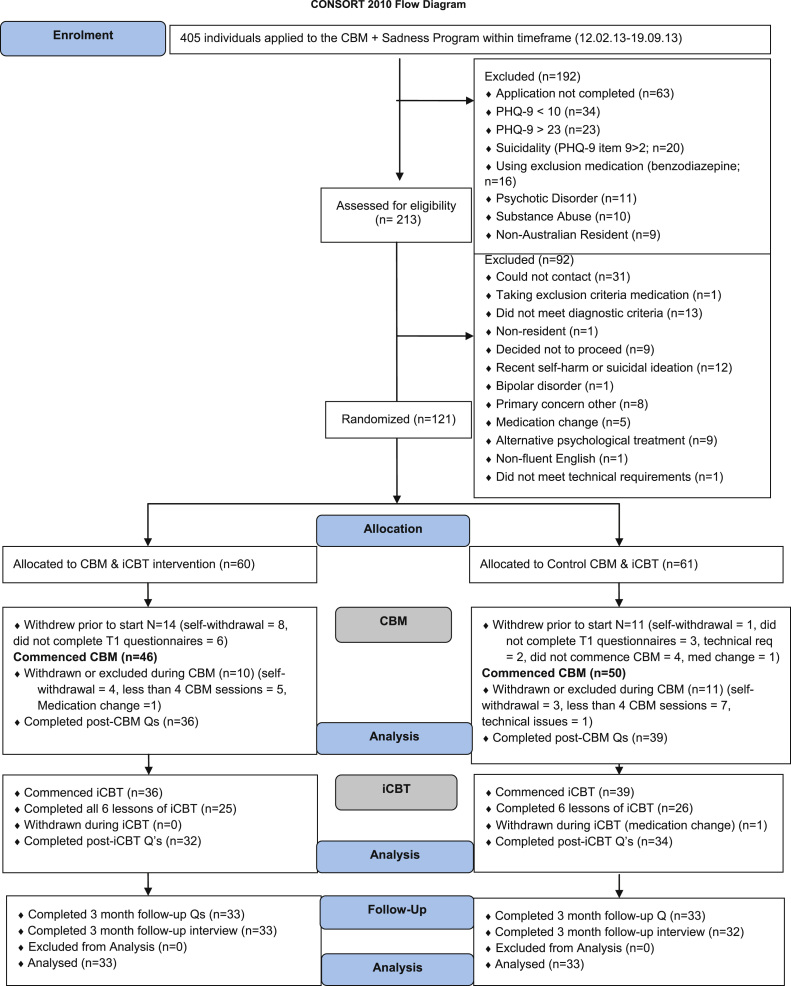


The minimum sample size for each group (alpha set at .05, power at .80) was identified as 29, following a power calculation using a between-group effect corresponding to Hedges׳s *g* of .66 (Lang et al., 2012), but an additional 10% was recruited to hedge against attrition. Recruitment was via the research arm of a not-for-profit clinical and research unit affiliated with St. Vincent׳s Hospital and the University of New South Wales, Australia that specializes in the development and evaluation of Internet-CBT. Applicants first completed online screening questionnaires assessing symptoms and demographic details (see Fig. 1: Participant Flowchart for inclusion and exclusion criteria). Applicants who passed this screening phase were telephoned for a diagnostic interview using the Mini International Neuropsychiatric Interview Version 5.0.0 (M.I.N.I; Sheehan et al., 1998) to determine whether they met diagnostic criteria for a major depressive episode. A registered psychologist trained in administration of the M.I.N.I conducted all interviews. Participants were informed of the study design and completed electronic informed consent prior to enrollment. Eligible participants accepted into the study were randomized based on an allocation sequence generated by an independent person not involved in the study via a true randomization process (www.random.org). Participants remained blind to group allocation. They were offered one entry into a draw for a gift card valued at $100 following completion of the 3-month follow-up. Clinicians conducting follow-up interviews were blind to patient information. The trial protocol (Williams et al., 2013a) was approved by the Human Research Ethics Committee of St. Vincent׳s Hospital and was prospectively registered on anzctr.org.au (ACTRN12613000139774) and clinicaltrials.gov (NCT01787513).

### 2.2 Interventions

#### 2.2.1 CBM program

Seven sessions of imagery CBM were completed daily online over the course of one week. Each session was approximately 15–20 min in duration (see Blackwell and Holmes (2010) and Williams et al. (2013b) for details). There were a total of 416 different training paragraphs, half recorded in a male voice and half recorded in a female voice and adapted to Australian cultural norms (e.g., reference to ‘snow’ was replaced with ‘rain’). Participants listened the training paragraphs, with the instruction to imagine themselves in the scenarios as if actively involved. The potential outcome of all scenarios was initially ambiguous and only resolved towards the end of the paragraph. In the positive condition all scenarios had a positive or benign resolution, thus establishing a learning contingency between the ambiguous start and the imagined resolution. For example: ‘You ask a friend to look over some work you have done. They come back with some comments, *which are all very positive*’ (positive resolution in italics). In the Control condition 50% of the scenarios had a positive resolution and 50% had a negative resolution (e.g., ‘They come back with some comments, *which are all critical*’), thus establishing no valence-specific training contingency. Adherence to the CBM program was monitored through the computer software. If a participant missed a session, a member of the research team sent an email reminder via the Virtual Clinic system or made telephone contact.

#### 2.2.2 iCBT – The Sadness Program

The Sadness Program has been evaluated in four previous trials (Perini et al., 2008, 2009; Titov et al., 2010; Watts et al., 2013), an effectiveness study (Williams and Andrews, 2013), and has been delivered as part of a routine clinical service in Australia since 2010 (see Andrews and Williams (In press)). The program consists of six online lessons representing best practice CBT, as well as regular homework assignments and access to supplementary resources delivered over the course of 10 weeks. Each lesson was designed using a cartoon narrative and included: psycho-education, behavioral activation, cognitive restructuring, graded exposure, problem solving, assertiveness skills, and relapse prevention. Patient queries throughout the program were primarily addressed by email contact from the Clinician (a doctoral level psychologist) or the research support officer. If clinically indicated, or if patients׳ K10 and/or PHQ9 scores deteriorated, the Clinician would make telephone contact.

### 2.3 Primary outcome measures

The *Patient Health Questionnaire-9* (PHQ9; Kroenke et al., 2001) and the *Beck Depression Inventory—Second Edition* (BDI-II; Beck et al., 1996) were administered to assess depression symptoms (*α*=.82 and .88, respectively). The *Ambiguous Scenarios Test for Depression* (AST-D; Berna et al., 2011) provided an index of interpretation bias (higher scores reflect a more positive bias). Two versions of the AST-D (*α*=.78 and .79) were presented in counterbalanced order (at baseline and following the 1-week CBM intervention). The *Kessler-10 Psychological Distress Scale* (K10; Kessler et al., 2002) measured non-specific psychological distress (modified in the current study to assess distress in the past week; *α*=.84). The PHQ9, BDI-II, and K10 were administered at baseline, following the 1-week CBM intervention, following the combined intervention (11 weeks), and at 3-month follow-up. Change in diagnostic status was assessed using the M.I.N.I (Sheehan et al., 1998) Depression Module administered via telephone at 3-month follow-up.

### 2.4 Secondary outcome measures

The following secondary measures were collected at baseline, after completion of the combined intervention, and at 3-month follow-up. The 12-item *World Health Organisation Disability Assessment Schedule* (WHODAS-II; World Health Organization) measured functional impairment and activity limitation (higher scores indicate greater disability; *α*=.83). The *Repetitive Thinking Questionnaire-10* (RTQ-10; McEvoy et al., 2010) measured perseverative negative thinking (higher scores indicate greater rumination; current sample *α*=.88). The *State Trait Anxiety Inventory-Trait Version* (STAI; Spielberger et al., 1983) was used to index trait anxiety (higher scores indicative of greater anxiety; *α*=.82). Comorbid Generalized Anxiety Disorder (GAD) and Social Phobia (SP) status were assessed at baseline and at 3-month follow-up with the M.I.N.I GAD and SP modules.

### 2.5 Additional measures

A *Treatment Expectancy* (adapted from Devilly and Borkovec (2000)) and *Outcomes Questionnaire* was administered at baseline (to assess how useful and logical participants expected the program to be) and following the combined intervention (to assess treatment satisfaction). The *Prospective Imagery Task* (PIT; Holmes et al., 2008; Stöber, 2000) was administered at baseline to check that groups were comparable for imagery ability (*α*=.85 for both the positive and negative subscales). The *Skills of Cognitive Therapy-Patient Version* (SoCT-P; Jarrett et al., 2011; *α*=.86) was administered following the combined intervention to assess participants use of cognitive strategies (higher scores reflect greater patient skill in applying cognitive therapy principles and coping strategies) and the association with depression symptom reduction.

### 2.6 Statistical methods

Significance testing of baseline group differences was conducted using ANOVA and *χ*^2^ where the variables consisted of nominal data. Efficacy analyses were conducted via mixed modeling using restricted maximum likelihood (REML) estimation. A linear mixed model for each outcome measure was implemented using the MIXED procedure with a random intercept for subject. As the random effect had one level, a variance components (VC) covariance structure was specified to model the covariance structure of the intercept (with the exception of the model for AST-D which required a diagonal structure). The effect of Group, Time, and the Group by Time interaction term were entered in each model. The between-group differences of interest were tested via pairwise contrasts provided by the EMEANS command^1^. Per protocol analyses of the primary hypotheses using data from participants who completed all 7 sessions of the CBM program were also conducted. Conservative effect size estimates were calculated between-group (Hedges *g*, using the pooled standard deviation and adjusting for sample size) and within-group (Cohen׳s *d*, adjusting for the repeated measures correlation using formula *d*=*t*⁎SQRT[(2(l−*r*)/*n*] (Dunlap et al., 1996)). Consistent with previous research (Vernmark et al., 2010; Williams et al., 2013b), clinically significant change was defined as high-end state functioning (BDI-II score<14) combined with a total score reduction greater than the reliable change index score (RCI) of 7.16 (Jacobson and Truax, 1991). Due to the high rate of comorbid anxiety disorders, clinically significant change was also calculated for the STAI-T based on a RCI of 7.86 in combination with a final score below the recommended cut-score of 45.7 (Fisher and Durham, 1999). Analyses were performed in SPSS version 22.

## 3 Results

### 3.1 Group characteristics and adherence

The final sample (after protocol specified exclusions and withdrawals^2^) included 36 participants (69% female) randomized to the positive condition and 39 participants (77% female) randomized to the control condition. There were no group differences in any of the demographic variables, all *p*>.05 (see Table 1) or in baseline measures or indices of treatment adherence, all *t*<1.55, all *p*>.05 (see Tables 1 and 2). Similar numbers completed all 7 sessions of CBM (positive condition: *n*=21, 58%; control: *n*=25, 64%) and all 6 lessons of iCBT (positive condition: *n*=25, 69%; control: *n*=26, 67%). A small number of participants completed zero iCBT lessons (positive condition: *n*=4, 11%; control: *n*=5, 13%).

### 3.2 Primary outcomes

Table 2 reports the results of the mixed-effects models. For depression symptoms there was a main effect of Time for the PHQ9, *F*(3, 205.59)=29.50, *p*<.001, and the BDI-II, *F*(3, 202.90)=52.22, *p*<.001. There were no significant main effects of Group or Time by Group interactions, all *F*<2, all *p*>.05. For interpretation bias there was a main effect of Time for the AST-D, *F*(1, 156.60)=24.24, *p* <.001, indicating a significant increase in positive interpretations in both groups. The main effect of Group, and the Time by group interaction, was not significant, *F*<1, *p*>.05.

### 3.3 Secondary outcomes

For psychological distress there was a main effect of Time for the K10 (collected at all 4 time points-see Table 2), *F*(3, 202.42)=54.81, *p*<.001. For all remaining secondary outcome measures (WHODAS, RTQ, STAI) separate linear mixed models were calculated with measurement occasion (3 time points) entered with a random intercept for subject. For each secondary outcome there was a main effect of Time, all *F* (2, 130.85–133.02)=37.56–70.75, all *p*<.001. Observed means and effect sizes are reported in Tables 2 and 3. There were no main effects of Group or Time by Group interactions for any of the secondary outcome measures.

### 3.4 Per protocol analyses

The per protocol sample consisted of participants (*n*=46) who completed all 7 CBM sessions (i.e., our pre-defined per protocol criterion; Williams et al., 2013a, 2013b). There were no differences between CBM completers and non-completers within each condition on baseline measures, all *t*<3 all *p*>.05. The only exception was lower mean scores on the AST-D in CBM completers in the control condition (relative to CBM non-completers in the Control conditions, *p*=.005, *ns* after adjustment for multiple comparisons). Importantly, in the per protocol sample there were no differences between conditions in any baseline measures. Table 4 reports the results of the main analyses.^3^ For the PHQ9 there was a main effect of Time, *F*(3, 124.59)=17.45, *p*<.001, but no overall Time by Group interaction, *p*=.10.The between-group differences were significant in favor of the positive condition following CBM and iCBT, *p*<.05. For the BDI-II there was a main effect of Time, *F*(3, 122.47)=32.39, *p*<.001, that was qualified by a significant interaction of Time by Group, *F*(3, 122.47)=4.28, *p*<.01. The between-group difference in favor of the positive condition was significant following CBM, *p*<.05, but not following iCBT, *p*=.08. For the AST-D only the main effect of Time was significant, *F*(1, 85.30)=26.49, *p*<.001. The between-group difference was not significant. For the K10 there was a main effect of Time, *F*(3, 122.88)=37.72, *p*<.001, and Group, *F*(1, 43.39)=4.19, *p*<.05, that was qualified by a significant Time by Group interaction, *F*(3, 122.88)=4.40, *p*<.05. The between-group differences in favor of the positive condition were significant following CBM and iCBT, *p*<.01.

### 3.5 Primary and secondary outcomes at 3-month follow-up

Gains were maintained for all outcome measures collected at the 3-month follow-up (i.e., there were no significant changes from post to follow-up on any measures), with no between-group differences (see Tables 2 and 3).^4^

### 3.6 Clinically significant change

Following the 1-week CBM phase, 61% of patients in the positive condition evidenced clinically significant change on the BDI-II compared to 44% in the control condition. Clinically significant change following iCBT was 69% in the positive condition and 50% in the control condition. The difference at both time points was not significant, *χ*^2^<3.5, *p*>.05. Interestingly, mean SoCT scores collected following the combined intervention were inversely correlated with depression scores (PHQ9 *r*=−.63, BDI-II *r*=−.59, *p*<.001) and were significantly higher in participants evidencing clinically significant change (*M*=27.65, *SD*=5.55) relative to those who did not (*M*=24.34, *SD*=5.26), *t*(64)=2.38, *p*<.05. Clinically significant change in anxiety scores (STAI) following the combined intervention was observed for 38% of the positive condition compared to 18% in the control condition, *χ*^2^ (1, 66)=3.26, *p=*.07.

### 3.7 Diagnostic status at follow-up

Diagnostic interviews were conducted for all 33 participants in the positive condition and 32/33 participants who completed the 3-month follow-up questionnaires in the control condition. 52% (*n*=17) of the positive condition and 56% (*n*=18) of the control condition (after they had completed the identical iCBT program for depression) no longer met diagnostic criteria for any of the disorders assessed at baseline (MDD, GAD, SP). 30% (*n*=10) of the positive condition and 19% (*n*=6) of the control condition met criteria for MDD. Comorbidity for the positive condition was as follows: GAD only (*n*=5, 15%), SP only (*n*=1, 3%), MDD/GAD (*n*=3, 9%), MDD/SP (*n*=1, 3%), and MDD/GAD/SP (*n*=5, 16%). Comorbidity for the control condition was as follows: GAD only (*n*=8, 25%), MDD/GAD/SP (*n*=5, 16%).

### 3.8 Post-hoc analyses of anhedonia

For the BDI-II anhedonia subscale (per protocol sample, following Blackwell et al. (2015)) there was a main effect of time, *F*(1,44)=18.14, *p*<.001 that was qualified by a significant Time by Group interaction, *F*(1,44)=5.04, *p*<.05. Anhedonia scores decreased significantly in the positive condition from pre-CBM (*M*=3.33, *SD*=1.19) to post-CBM (*M*=2.42, *SD*=1.20) corresponding to a medium effect (Cohen׳s *d*=.57, 95% CI=−.01 to 1.15), *t*(73)=4.41. The decrease in anhedonia scores in the control condition from pre-CBM (*M*=3.32, *SD*=1.65) to post-CBM (*M*=3.04, *SD*=1.96) was not significant, nor was the between-group effect, *F*<1.5, *p*>.05.

### 3.9 Treatment acceptability

Treatment acceptability and program evaluation ratings are reported in Table 1. There were no between-group differences in ratings, all *p*>.05.

## 4 Discussion

The current RCT sought to replicate the initial successful application of imagery CBM delivered via the Internet without face-to-face contact, and to establish whether positive imagery CBM is superior to a CBM control version when delivered to participants with a current major depressive episode. Similar to our previous findings (Williams et al., 2013b) in the full sample we found moderate-large within-group ESs for reductions in our primary measures of depression (PHQ9, BDI-II) and secondary measure of psychological distress (K10) in participants assigned to the positive condition. However, contrary to prediction, moderate-large within-group ESs were also observed for participants in the control condition and there were no between-group differences in outcomes in the full sample.

The lack of a differential effect between the two conditions raises a number of important considerations. As previously noted (Williams et al., 2013a), we chose a control condition that would be closely matched to our active intervention in regards to the structure of the task, imagery generation, repeated practice of a computer task, and contact with the research team. The standard structure of the ‘control’ condition employed in CBM interpretation bias studies has consisted of a 50/50 ratio of negative to positive disambiguated training scenarios based on the assumption that a specific learning contingency is not established under these conditions. Yet, experimental and clinical studies have recently converged to suggest that the crucial ‘ingredients’ of the imagery CBM task are the presence of initial emotional ambiguity in the training scenarios and the use of imagery (Clarke et al., 2014a; Torkan et al., 2014). Therefore it is possible that the control variant employed in the current trial represented a smaller ‘dose’ of CBM positive training, thus leading to positive therapeutic effects observed in our CBM control group. Considering the apparent importance of these two features the most appropriate control conditions in future trials may therefore contain neither emotional ambiguity nor use of imagery, in order to reduce the extent to which a control condition contains too many of the ‘active ingredients’ of the CBM intervention.

The lack of between-group differences could also be attributable to dilution of effects in the full sample. Further, having the intervention delivered entirely remotely may have reduced levels of task fidelity (and thus a smaller signal to noise ratio in the data) compared to studies in which training to task requirements was provided in an initial face-to-face session (e.g., Lang et al., 2012; Torkan et al., 2014). It is also possible that the sample recruited was one in which it would have been particularly difficult to detect between-group differences: Blackwell et al. (2015) found superiority of a four-week Internet-delivered imagery CBM over an active control only for participants with fewer than 5 episodes of depression, which corresponds to only 16% of the sample in the current RCT.

It is interesting that we found greater improvement in the control condition than might be expected on the basis of previous smaller-scale clinical studies (a mean reduction on the BDI-II from pre to post-control intervention of 7.69 in the current trial, compared to .54 for Lang et al., 2012 or 3.93 for Torkan et al., 2014). The control condition in the current study was similar to that used by Lang et al. (2012) in that the only difference from the positive condition was that half of the training stimuli were resolved positively and half were resolved negatively (although the training paradigm in the current study was only one of three used by Lang et al. (2012)). However, in the context of a clinical trial in which participants are treatment-seeking individuals believing they may be receiving an intervention to change their thinking styles (which was not the case for Lang et al. (2012), which was an experimental study), such a control condition may perform differently (cf. Blackwell et al., 2015).

It is important to note that a shift in interpretation bias, towards more positive interpretations of ambiguous information, was observed in both conditions. As the induced shift in processing ambiguous information at least partially mediated the effect of CBM training on depression symptoms in our previous RCT (Williams et al., 2013b), it therefore may not be surprising that equivalent reductions in primary symptom measures occurred in both groups. The similar change on the AST-D in the per protocol sample does not allow us to conclude that the differential symptom outcome was mediated by changes in interpretive bias, and thus it may be other factors, such as receiving more practice in generating specifically positive imagery that contributed to the additional benefit on symptoms of depression conferred by the positive condition. Greater specificity in assessing bias change via inclusion of a measure indexing a wider range of depressogenic cognitions (Rohrbacher and Reinecke, 2014), or objective measurement of bias change, such as evidence of violations of expectancy gathered by ERP responses from EEG studies (Moser et al., 2012) might serve as useful additions in future studies.

A strength of the current study is the inclusion of diagnostic interviews conducted at 3-month follow-up. The majority of participants in both groups no longer met diagnostic criteria for any disorder that was assessed at baseline after completing the same iCBT program for depression. Not only did diagnostic status for the primary disorder of MDD change significantly, but the percentage of comorbid GAD and Social Phobia also reduced. The combined intervention (in both conditions) also resulted in significant reductions in secondary outcome measures including disability, repetitive negative thinking and trait anxiety, corresponding to medium to large effect sizes.

In our per protocol sample (those who completed all seven sessions of CBM and thus received the intended ‘dose’ of the intervention) we did find significant between-group differences from pre to post-CBM, which remained significant after the 10-week iCBT program for the PHQ9 and K10. Further, and consistent with a recent RCT of a four-week positive imagery CBM intervention, in post-hoc analyses we found a significant advantage for the positive CBM intervention over the control condition for anhedonia. The superiority of the positive over control condition on symptom outcomes over the one-week intervention in the per protocol sample is consistent with the earlier small clinical studies that have shown superiority over an active control. We note that the high rate of adherence in these previous studies is similar to the per protocol requirements (58% completed all 7 positive CBM sessions) in the current study.

We have previously speculated that CBM may serve a preparatory function for engagement in subsequent therapy, perhaps by boosting motivation to engage in the schedule imposed by (Internet) CBT (e.g. via boosting positive mood or behavioral engagement directly; cf. Pictet et al., 2011); or by facilitating success with certain CBT techniques (e.g., generating positive alternative thoughts or anticipating success from behavioral tasks). Given the minimal differences between the two conditions during the CBM phase, it is encouraging to find differential outcomes 10 weeks later after an identical iCBT program. While there was no evidence for superiority of the positive condition in the longer-term (i.e., 3 month follow-up), accelerating recovery is a useful goal in itself. Qualitative feedback from our trial participants suggests that CBM might facilitate cognitive re-appraisal even in the absence of the explicit guided instruction used in CBT (e.g., ‘*The CBM program made me very aware of my negative thinking…. I learned to not always expect the worst*’). This raises the hypothesis that people who have previously tried CBT but struggle with explicit cognitive challenging tasks may benefit from a CBM module that targets this process implicitly through training. The reduction in anhedonia over the one-week positive imagery CBM observed in our per protocol sample is encouraging in adding further support to the suggestion by Blackwell et al. (2015) that imagery CBM may be a useful tool for treating anhedonia in depression.

The current findings need to be considered in light of a number of limitations. The absence of another control comparator makes it difficult to attribute the beneficial effects solely to the intervention. In a previous RCT employing highly similar recruitment and delivery procedures (Williams et al., 2013b), we did not observe any significant changes in a wait-list control group following the same 1-week CBM intervention phase where some non-specific factors (e.g., contact with the research team) were matched. We cannot exclude the possibility that symptom reductions reflected the operation of a placebo effect or that clinical response measured at 3-month follow-up was a result of natural fluctuations in symptoms across time. However, the magnitude of the reductions across all symptom measures suggests this explanation is unlikely. Although the sample was comprised of individuals meeting diagnostic criteria for depression (many of whom presented with a history of depressive episodes and clinical comorbidities), it is unknown whether the current results would generalize to more severe patients not taking part in a research trial or to those with other Axis I or II disorders not assessed in the current study. Results of the per protocol analyses must also be interpreted with caution given the reduced sample size in both groups after selecting out those who completed all 7 CBM sessions, and the fact that per protocol samples may lose some of the benefits of randomization, leading to increased risk of bias (e.g. participant self-selection; Moher et al., 2010). Tracking the trajectory of change across CBM sessions would inform our understanding of the minimal ‘dose’ required to lead to therapeutic change and therefore enable better understanding of what constitutes an intervention ‘completer’.

In summary, although results did not support our primary hypotheses, the lack of differential outcomes could possibly be attributable to the active ingredients contained in our control comparator. Inclusion of an alternative control group would enable a more rigorous evaluation of this proposal. Results are encouraging for both the standalone benefits of the one-week imagery CBM module and the combined CBM and iCBT intervention, when all 7 sessions of CBM are completed (as in our per protocol sample). Given that between-group differences were found in the per protocol sample but not in the full sample, enhancing adherence to the CBM sessions or identifying people who may be less likely to complete (and therefore benefit) should be a focus of future research. Results will inform further clinical trials of the combined intervention and basic research investigating the range of potential treatment mechanisms. How best to combine CBM and iCBT approaches to optimize the overall treatment package is an important question for further research.

## Financial support

Alishia D. Williams is supported by an Australian National Health and Medical Research Council (NHMRC) Fellowship (630746). Portions of this research were supported by a University of New South Wales (PS16798), Australia Faculty of Medicine grant awarded to Alishia D. Williams and a Bupa Health Foundation (RG124613) grant awarded to Alishia D. Williams, Emily A. Holmes, Simon E. Blackwell, and Gavin Andrews. Emily A. Holmes and Simon E. Blackwell are supported by the Medical Research Council (United Kingdom) intramural programme (MC-A060-5PR50) and received support from the Lupina Foundation. Emily A. Holmes is also supported by a Wellcome Trust Clinical Fellowship (WT088217), and the National Institute for Health Research (NIHR) Oxford Biomedical Research Centre Programme. The views expressed are those of the author(s) and not necessarily those of the NHS, the NIHR or the Department of Health. The study sponsor and funders had no role in the study design. Open Access publication charges for this article was provided by the UK Medical Research Council.

## Conflict of interest

Professor Gavin Andrews is the Director of the Clinical Research Unit for Anxiety and Depression (CRUfAD) and the This Way Up (https://thiswayup.org.au) service in Australia that provides internet-based CBT programs to practitioners and patients.

## Figures and Tables

**Table 1:** Demographic and baseline characteristics, treatment expectancy, and adherence and treatment acceptability by condition.

	Positive condition	Control condition
	(*n*=36)	(*n*=39)

Gender		
Female	25 (69%)	30 (77%)
Male	11 (31%)	9 (23%)
Age	43.94 (10.80)	39.86 (11.93)
Australia/New Zealand Born	29 (81%)	31 (79%)
English as Primary Language	34 (94%)	36 (92%)
Marital Status		
Single	8 (22%)	16 (41%)
Married/Defacto	18 (50%)	12 (31%)
Separated/Divorced	5 (14%)	9 (23%)
Widowed	5 (14%)	2 (5%)
Work Status		
Employed	17 (48%)	22 (56%)
Unemployed	3 (8%)	3 (8%)
Retired	3 (8%)	2 (5%)
Disability Pension	3 (8%)	5 (13%)
Other	10 (28%)	7 (18%)
Education		
Year 10/12 Certificate	7 (20%)	12 (31%)
Trade/Technical Certificate	8 (22%)	4 (10%)
Undergraduate Diploma	6 (17%)	6 (15%)
Bachelor Degree	12 (33%)	14 (36%)
Master/Doctoral Degree	3 (8%)	3 (8%)
Current Antidepressant Medication		
No	18 (50%)	18 (46%)
Yes	18 (50%)	21 (54%)
Comorbidity		
None	14 (39%)	9 (23%)
Generalized Anxiety Disorder and Social Phobia	12 (33%)	12 (31%)
Generalized Anxiety Disorder	6 (17%)	13 (33%)
Social Phobia	4 (11%)	5 (13%)
Age First Depressive Episode	22.13 (10.87)	21.05 (11.30)
Number of Previous Depressive Episodes		
0–1	1 (3%)	4 (10%)
2–3	4 (11%)	3 (8%)
≥ 4	31 (86%)	32 (82%)

	**[*****M*****,*****SD*****]**	**[*****M*****,*****SD*****]**
Treatment Expectancy		
Logical	3.22 (1.14)	3.56 (.96)
Useful	3.30 (.89)	3.11 (.85)
Number of CBM sessions completed	6.33 (.95)	6.30 (1.07)
Number of iCBT lessons completed	4.86 (2.07)	4.69 (2.20)
Prospective Imagery Task (PIT)		
Positive subscale	23.66 (6.78)	23.30 (7.19)
Negative subscale	32.83 (7.42)	33.05 (9.08)
Feedback for CBM program^a^		
Satisfied with # of sessions	52% (*n*=19)	51% (*n*=20)
Reduce # of sessions	42% (*n*=15)	41% (*n*=16)
Increase # of sessions	5% (*n*=2)	8% (*n*=3)
Treatment satisfaction^a^		
Dissatisfied/neutral	25% (*n*=8)	29% (*n*=10)
Mostly/very satisfied	75% (*n*=24)	71% (*n*=24)
Program quality^a^		
Unsatisfactory	3% (*n*=1)	3% (*n*=1)
Satisfactory	13% (*n*=4)	17% (*n*=6)
Good/Excellent	84% (*n*=27)	80% (*n*=27)
Confidence recommending to friend	7.18 (1.95)	7.11 (2.04)
Access to iCBT content post-treatment	2.15 (.93)	2.18 (.72)

*Note*: Number of previous depressive episodes based on participant self-report. Confidence recommending the program to a friend with depression rated 0–10. Access to iCBT content post-treatment=frequency participants accessed the iCBT material after program completion (assessed at 3-month follow-up).

a % based on positive condition *n*=32; control condition *n*=34.

**Table 2:** Observed means (standard deviations) for measures and within-group effect sizes in the full sample.

	Baseline Mean (SD)	Post-CBM Mean (SD)	Within *t*(df) *r*	Between *t*(df)	Post-iCBT Mean (SD)	Within *t*(df) *r*	Between *t*(df)	3 Month Mean (SD)	Within *t*(df) *r*	Between *t*(df)
	T1	T2	ES (95% CI) T1, T2	ES (95% CI) T1, T2	T3	ES (95% CI) T1, T3	ES (95% CI) T1, T3	T4	ES (95% CI) T3, T4	ES (95% CI) T3, T4
PHQ9 Positive	16.22 (5.34)	8.97 (5.95)	6.09 (3202.30)⁎⁎*r*=.38	1.27 (1219.89)	8.68 (6.56)	5.96 (3206.16)⁎⁎*r*=.49	.92 (1233.59)	9.21 (6.62)	.32 (3204.78) *r*=.63	.92 (1233.59)
			**1.13** (.66–1.59)	.27 (−.17 to .73)		**1.06** (.56–1.55)	.26 (−.22 to .74)		.04 (−.44 to .52)	.17 (−.31 to .65)
PHQ9 Control	15.33 (4.89)	10.74 (6.56)	4.01 (3202.30)⁎⁎*r*=.53		10.35 (6.08)	4.28 (3206.96)⁎⁎*r*=.15		8.06 (6.19)	1.59 (3207.72) *r=*.27	
			**.62** (.17–1.06)			**.96** (.48–1.43)			.33 (−.15 to .81)	
BDI-II Positive	31.05 (8.48)	20.36 (7.82)	5.72 (3200.72)⁎⁎*r*=.51	1.47 (1162.13)	15.50 (11.88)	7.76 (3203.29)⁎⁎*r=*.65	1.15 (1178.72)	17.33 (12.70)	.91 (3202.24) *r=*.60	1.15 (1178.72)
			**.94** (.47–1.40)	.34 (−.11 to .80)		**1.14** (.64–1.63)	.32 (−.15 to .81)		.14 (−.34 to .62)	.25 (−.23 to .73)
BDI-II Control	31.84 (10.93)	24.15 (13.10)	4.28 (3200.72)⁎⁎*r*=.65		19.23 (10.68)	6.79 (3203.87)⁎⁎*r*=.42		14.06 (12.26)	2.03 (3203.19) *r=*.37	
			**.57** (.12–1.01)			**1.25** (.77–1.72)			.39 (−.09 to .87)	
AST-D Positive	3.75 (.95)	4.65 (.89)	3.83 (1141.48)⁎⁎*r*=.38	.97 (173.00)						
			**.71** (.24–1.17)	.22 (−.23 to .67)						
AST-D Control	3.59 (1.18)	4.45 (.91)	3.78 (1141.48)⁎⁎*r*=.29							
			**.72** (.27–1.16)							
K10 Positive	30.36 (6.54)	25.13 (6.48)	4.54 (3200.37)⁎⁎*r*=.65	1.23 (1155.05)	19.56 (6.87)	8.61 (3202.79)⁎⁎*r*=.49	1.79 (1171.22)	22.12 (7.53)	2.01 (3201.78) *r=*.59	1.79 (1171.22)
			**.63** (.16–1.09)	.30 (−.14 to .76)		**1.53** (1.03–2.02)	.50 (.01–.99)		.31 (−.17 to .79)	.11 (−.37 to .59)
K10 Control	31.56 (6.30)	27.15 (6.50)	3.99 (3200.37)⁎⁎*r*=.71		23.11 (7.09)	7.32 (3203.33)⁎⁎*r*=.48		21.27 (7.73)	1.04 (3202.67) *r=*.50	
			**.48** (.03–.92)			**1.28** (.69–1.64)			.18 (−.30 to .66)	

*Note.* T1=Baseline, T2=Post-CBM, T3=Post-CBM+ iCBT, T4=3-month follow-up. PHQ9=Patient Health Questionnaire-9 item; BDI-II=Beck Depression Inventory-2nd Edition; K10=Kessler Distress Scale – 10 item; AST-D=Ambiguous Scenarios Test for Depression. Within-group ES=Cohen׳s *d* based on the formula: *d*=t*SQRT[2(l−*r*)/*n*]. *N* T1: Positive=36, Control=39; T2: Positive=36, Control=39; T3: Positive=32, Control=34; T4: Positive=33, Control=33.

⁎⁎ *p*<.001.

**Table 3:** Observed means (standard deviations) for secondary measures and within and between-group effect sizes in full sample.

	Baseline Mean (SD)	Post-iCBT Mean (SD)	Within *t*(df)r	Between *t*(df)	3 Month Mean (SD)	Within *t*(df) *r*	Between *t*(df)
	T1	T3	ES (95% CI) T1, T3	ES (95% CI) T1, T2	T4	ES (95% CI) T3, T4	ES (95% CI) T3, T4
WHODAS Positive	32.58 (9.30)	23.54 (9.16)	5.32 (2130.92)⁎⁎*r=*.43	.71 (1156.65)	24.87 (9.24)	.74 (2129.09) *r=*.78	1.14 (1156.56)
			**.82** (.32–1.31)	.17 (−.30 to .65)		.08 (−.40 to .56)	.28 (−.20 to .76)
WHODAS Control	33.05 (9.29)	25.17 (9.15)	4.79 (2131.88)⁎⁎*r=*.48		22.24 (9.12)	1.71 (2130.42) *r=*.44	
			**.83** (.35–1.30)			.21 (−.27 to .69)	
RTQ Positive	40.67 (10.08)	28.84 (9.45)	7.09 (2133.37)⁎⁎*r=*.56	1.02 (1149.89)	28.13 (9.47)	.41 (2131.62) *r=*.83	.81 (1149.80)
			**1.17** (.49–1.66)	.25 (−.23 to .73)		.04 (−.44 to .52)	.19 (−.28 to .68)
RTQ Control	42.07 (9.60)	31.26 (9.44)	6.71 (2134.27)⁎⁎*r=*.34		30.03 (9.41)	.71 (2132.77) *r=*.34	
			**1.32** (.84–1.79)				
						.14 (−.34 to .22)	
STAI Positive	61.36 (10.56)	51.71 (10.41)	5.01 (2133.10)⁎⁎*r=*.57	1.22 (1158.08)	51.98 (10.44)	.12 (2131.29) *r=*.63	.04 (1157.99)
			**.81** (.31–1.30)	.29 (−.18 to .78)		.01 (−.47 to .49)	.01 (−.47 to .49)
STAI Control	63.61 (10.54)	54.86 (10.37)	4.70 (2134.04)⁎⁎*r=*.22		51.86 (10.33)	1.54 (2132.59) *r=*.52	
			**1.01** (.53–1.48)			.26 (−.22 to .74)	

*Note.* T1=Baseline, T3=Post-CBM+iCBT, T4=3 month follow-up. WHODAS=World Health Organization Disability Assessment Schedule-II; RTQ=Repetitive Thinking Questionnaire. STAI-T=State Trait Anxiety Inventory-Trait; Within-group ES=Cohen׳s *d* based on the formula: *d*=t*SQRT[2(l−*r*)/*n*]. *N* T1: Positive=36, Control=39; T3: Positive=32, Control=34; T4: Positive=33, Control=33.

⁎⁎ *p*<.001.

**Table 4:** Observed means (standard deviations) and within and between-group effect sizes for the CBM per protocol sample.

	Baseline Mean (SD)	Post-CBM Mean (SD)	Within *t*(df) *r*	Between *t*(df)	Post-iCBT Mean (SD)	Within *t*(df) *r*	Between *t*(df)	3 Month Mean (SD)	Within *t*(df) *r*	Between *t*(df)
	T1	T2	ES (95% CI) T1, T2	ES (95% CI) T1, T2	T3	ES (95% CI) T1, T3	ES (95% CI) T1, T3	T4	ES (95% CI) T3, T4	ES (95% CI) T3, T4
PHQ9 Positive	15.80 (5.51)	8.14 (6.35)	4.85 (3122.78)⁎⁎ *r*=.37	2.12 (1137.28)⁎	7.20 (4.94)	5.32 (3124.01)⁎⁎ *r*=.58	1.98 (1141.07)⁎	9.68 (6.14)	1.48 (3124.86) *r=*.52	.14 (1109.19)
			**1.18** (.57–1.78)	**.55** (−.03 to 1.13)		**1.09** (.46–1.71)	**.67** (.06–1.27)		.25 (−.23 to .73)	.09 (−.38 to .58)
PHQ9 Control	16.00 (4.97)	11.92 (7.02)	2.82 (3122.78) ns *r*=.61		11.04 (6.06)	3.42 (3124.87)⁎ *r*=.03		9.05 (6.64)	1.08 (3125.15) *r=*.27	
			.49 (−.06 to 1.04)			**.99** (.40–1.57)			.22 (−.21 to .75)	
BDI-II Positive	30.42 (8.04)	19.71 (8.22)	4.43 (3121.32)⁎⁎ *r*=.59	2.41 (1,96.79)⁎	14.20 (11.47)	6.53 (3122.17)⁎⁎ *r=*.35	1.75 (1101.82)	19.05 (11.68)	2.07 (3121.82) *r=*.44	1.27 (191.90)
			**.87** (.26–1.47)	**.70** (.11–1.28)		**1.66** (1.02–2.29)	.57 (−.03 to 1.17)		.38 (−.04 to .92)	.34 (−.14 to .83)
BDI-II Control	33.32 (10.66)	27.60 (12.78)	2.58 (3121.32) *r*=.69		20.73 (10.69)	5.73 (3122.76)⁎ *r=*.35		14.75 (12.94)	1.84 (3122.67) *r=*.37	
			.40 (−.15 to .95)			**1.36** (.77–1.94)			.35 (−.13 to .83)	
AST-D Positive	3.69 (1.05)	4.63 (.90)	3.11 (185.30)⁎ *r*=.19	.97 (173.00)						
			**.86** (.30–1.41)	**.27** (−.31 to .85)						
AST-D Control	3.21 (1.07)	4.38 (.88)	4.21 (185.31)⁎⁎ *r*=.12							
			**1.11** (.55–1.66)							
K10 Positive	30.66 (5.73)	23.42 (4.79)	4.81 (3121.60)⁎⁎ *r*=.72	2.67 (1103.77)⁎⁎	18.35 (5.59)	7.87 (3122.53)⁎⁎ *r*=.60	3.10 (1111.21)⁎⁎	23.21 (6.48)	3.10 (3122.17) *r=*.29	.16 (188.56)
			**.78** (.17–1.38)	**.88** (.29–1.46)		**1.57** (.94–2.19)	**.90** (.29–1.50)		.64 (.15–1.12)	.14 (−.11 to .62)
K10 Control	32.60 (6.25)	29.48 (8.02)	2.26 (3121.60) *r*=.74		24.08 (6.78)	6.11 (3123.17)⁎⁎ *r*=.25		22.15 (7.98)	.84 (3123.12) *r=*.53	
			.32 (−.23 to .87)			**1.56** (.97–2.14)			.14 (−.34 to .62)	

*Note.* Between-group significance values: ⁎⁎*p*<.01, ⁎*p*<.05. Bonferroni adjusted within-group significance values ⁎⁎*p<*.001, ⁎*p*<.01. T1=Baseline, T2=Post-CBM, T3=Post-CBM+iCBT, T4=3-month follow-up. PHQ9=Patient Health Questionnaire-9 item; BDI-II=Beck Depression Inventory- 2nd Edition; K10=Kessler Distress Scale – 10 item; AST-D=Ambiguous Scenarios Test for Depression. Within-group ES=Cohen׳s *d* based on the formula: *d*=t*SQRT[2(l−*r*)/*n*]. *N* T1: Positive=21, Control=25; T2: Positive=21, Control=25; T3: Positive=20, Control=23; T4: Positive=19, Control=20.

## Footnotes

1 All models included Time as a fixed effect across all four indicated measurement time-points; therefore our hypotheses were addressed via the between-group contrasts rather than overall model effects.

2 The modified ITT analyses did not include participants who were formally withdrawn for reasons pre-specified in the trial protocol. All other participants were included, irrespective of adherence (see Footnote 4).

3 Due to a system error, in the control condition 8 participants completed 1 session and 5 participants completed 2 sessions of the positive CBM in place of the control CBM. The pattern of results remained unchanged when selecting out these participants from the modified ITT analyses.

4 Note that data collected at 3-month follow-up contained 9 participants (*n*=4 positive, *n*=5 control) who did not complete any iCBT lessons and 4 participants (*n*=2 each group) who did not supply data post-iCBT.

## References

[bib1] Andersson G. (2009). Using the internet to provide cognitive behaviour therapy. Behav. Res. Ther..

[bib2] Andrews G., Williams A.D. (2015). Up-scaling clinician assisted internet cognitive behavioural therapy (iCBT) for depression: a model for dissemination into primary care. Clin. Psychol. Rev..

[bib3] Beck A.T., Steer R.A., Brown G.K. (1996). Beck Depression Inventory—Manual.

[bib4] Berna C., Lang T.J., Goodwin G.M., Holmes E.A. (2011). Developing a measure of interpretation bias for depressed mood: an ambiguous scenarios test. Personal. Individ. Differ..

[bib5] Blackwell S.E., Browning M., Mathews A., Pictet A., Welch J., Davies J., Holmes E.A. (2015). Positive imagery-based cognitive bias modification as a web-based treatment tool for depressed adults: a randomized controlled trial. Clin. Psychol. Sci..

[bib6] Blackwell S.E., Holmes E.A. (2010). Modifying interpretation and imagination in clinical depression: a single case series using cognitive bias modification. Appl. Cogn. Psychol..

[bib7] Butler G., Mathews A. (1983). Cognitive processes in anxiety. Adv. Behav. Res. Ther..

[bib8] Clarke P.J., Nanthakumar S., Notebaert L., Holmes E.A., Blackwell S.E., Macleod C. (2014). Simply imagining sunshine, lollipops and rainbows will not budge the bias: the role of ambiguity in interpretive bias modification. Cogn. Ther. Res..

[bib9] Clarke P.J., Notebaert L., MacLeod C. (2014). Absence of evidence or evidence of absence: reflecting on therapeutic implementations of attentional bias modification. BMC Psychiatry.

[bib10] Devilly G.J., Borkovec T.D. (2000). Psychometric properties of the credibility/expectancy questionnaire. J. Behav. Ther. Exp. Psychiatry.

[bib11] Dunlap W.P., Cortina J.M., Vaslow J.B., Burke M.J. (1996). Meta-analysis of experiments with matched groups or repeated measures designs. Psychol. Methods.

[bib12] Fisher P.L., Durham R.C. (1999). Recovery rates in generalized anxiety disorder following psychological therapy: an analysis of clinically significant change in the STAI-T across outcome studies since 1990. Psychol. Med..

[bib13] Holmes E.A., Craske M.G., Graybiel A.M. (2014). Psychological treatments: a call for mental-health science. Nature.

[bib14] Holmes E.A., Lang T.J., Deeprose C. (2009). Mental imagery and emotion in treatment across disorders: using the example of depression. Cogn. Behav. Ther..

[bib15] Holmes E.A., Lang T.J., Moulds M.L., Steele A.M. (2008). Prospective and positive mental imagery deficits in dysphoria. Behav. Res. Ther..

[bib16] Holmes E.A., Lang T.J., Shah D.M. (2009). Developing interpretation bias modification as a “cognitive vaccine” for depressed mood: imagining positive events makes you feel better than thinking about them verbally. J. Abnorm. Psychol..

[bib17] Holmes E.A., Mathews A., Dalgleish T., Mackintosh B. (2006). Positive interpretation training: effects of mental imagery versus verbal training on positive mood. Behav. Ther..

[bib18] Jacobson N.S., Truax P. (1991). Clinical significance: a statistical approach to defining meaningful change in psychotherapy research. J. Consult. Clin. Psychol..

[bib19] Jarrett R.B., Vittengl J.R., Clark L.A., Thase M.E. (2011). Skills of cognitive therapy (SoCT): a new measure of patients׳ comprehension and use. Psychol. Assess..

[bib20] Kessler R.C., Andrews G., Colpe L.J., Hiripi E., Mroczek D.K., Normand S.L., Zaslavsky A.M. (2002). Short screening scales to monitor population prevalences and trends in non-specific psychological distress. Psychol. Med..

[bib21] Kroenke K., Spitzer R.L., Williams J.B. (2001). The PHQ-9: validity of a brief depression severity measure. J. Gen. Intern. Med..

[bib22] Lang T.J., Blackwell S.E., Harmer C.J., Davison P., Holmes E.A. (2012). Cognitive bias modification using mental imagery for depression: developing a novel computerized intervention to change negative thinking styles. Eur. J. Personal..

[bib23] Macleod C. (2012). Cognitive bias modification procedures in the management of mental disorders. Curr. Opin. Psychiatry.

[bib24] Mathews A., Mackintosh B. (2000). Induced emotional interpretation bias and anxiety. J. Abnorm. Psychol..

[bib25] McEvoy P.M., Mahoney A.E., Moulds M.L. (2010). Are worry, rumination, and post-event processing one and the same? Development of the repetitive thinking questionnaire. J. Anxiety Disord..

[bib26] Moher D., Hopewell S., Schulz K., Montori V., Gotzsche P., Devereaux P., Altman D. (2010). CONSORT 2010 explanation and elaboration: updated guidelines for reporting parallel group randomised trials. Br. Med. J..

[bib27] Morina N., Deeprose C., Pusowski C., Schmid M., Holmes E.A. (2011). Prospective mental imagery in patients with major depressive disorder or anxiety disorders. J. Anxiety Disord..

[bib28] Moser J.S., Huppert J.D., Foa E.B., Simons R.F. (2012). Interpretation of ambiguous social scenarios in social phobia and depression: evidence from event-related brain potentials. Biol. Psychol..

[bib29] Perini S., Titov N., Andrews G. (2008). The climate sadness program of internet-based treatment for depression: a pilot study. E-J. Appl. Psychol..

[bib30] Perini S., Titov N., Andrews G. (2009). Clinician-assisted Internet-based treatment is effective for depression: randomized controlled trial. Aust. N. Z. J. Psychiatry.

[bib31] Pictet A., Coughtrey A.E., Mathews A., Holmes E.A. (2011). Fishing for happiness: the effects of generating positive imagery on mood and behaviour. Behav. Res. Ther..

[bib32] Rohrbacher H., Reinecke A. (2014). Measuring change in depression-related interpretation bias: development and validation of a parallel ambiguous scenarios test. Cogn. Behav. Ther..

[bib33] Sheehan D.V., Lecrubier Y., Sheehan K.H., Amorim P., Janavs J., Weiller E., Dunbar G.C. (1998). The Mini-International Neuropsychiatric Interview (M.I.N.I.): the development and validation of a structured diagnostic psychiatric interview for DSM-IV and ICD-10. J. Clin. Psychiatry.

[bib34] Simon G.E., Ludman E.J. (2009). It׳s time for disruptive innovation in psychotherapy. The Lancet.

[bib35] Spielberger C.D., Gorsuch R.L., Lushene R., Vagg P.R., Jacobs G.A. (1983). Manual for the State-Trait Anxiety Inventory.

[bib36] Stöber J. (2000). Prospective cognitions in anxiety and depression: replication and methodological extension. Cogn. Emot..

[bib37] Titov N., Andrews G., Davies M., McIntyre K., Robinson E., Solley K. (2010). Internet treatment for depression: a randomized controlled trial comparing clinician vs. technician assistance. PLoS One.

[bib38] Torkan H., Blackwell S.E., Holmes E.A., Kalantari M., Neshat-Doost H.T., Maroufi M., Talebi H. (2014). Positive imagery cognitive bias modification in treatment-seeking patients with major depression in Iran: a pilot study. Cogn. Ther. Res..

[bib39] Vernmark K., Lenndin J., Bjarehed J., Carlsson M., Karlsson J., Oberg J., Andersson G. (2010). Internet administered guided self-help versus individualized e-mail therapy: a randomized trial of two versions of CBT for major depression. Behav. Res. Ther..

[bib40] Watts S., Mackenzie A., Thomas C., Griskaitis A., Mewton L., Williams A., Andrews G. (2013). CBT for depression: a pilot RCT comparing mobile phone vs. computer. BMC Psychiatry.

[bib41] Williams A.D., Andrews G. (2013). The effectiveness of internet cognitive behavioural therapy (iCBT) for depression in primary care: a quality assurance study. PLoS One.

[bib42] Williams A.D., Blackwell S.E., Holmes E.A., Andrews G. (2013). Positive imagery cognitive bias modification (CBM) and internet-based cognitive behavioural therapy (iCBT) versus control CBM and iCBT for depression: study protocol for a parallel-group randomised controlled trial. BMJ Open.

[bib43] Williams A.D., Blackwell S.E., Mackenzie A., Holmes E.A., Andrews G. (2013). Combining imagination and reason in the treatment of depression: a randomized controlled trial of internet-based cognitive-bias modification and internet-CBT for depression. J. Consult. Clin. Psychol..

[bib44] World Health Organization (2008). The Global Burden of Disease: 2004 Update.

[bib1000] World Health Organization Disability Assessment Schedule II (WHODAS II) 〈http://www.who.int/icidh/whodas/〉

