# Positive imagery cognitive bias modification (CBM) and internet-based cognitive behavioural therapy (iCBT): A randomized controlled trial

**DOI:** 10.1016/j.jad.2015.08.020

**Published:** 2016-01-15

There was an error in Section 3.6. The published text reports the percentages for clinically reliable change, not clinically reliable and signiifcant change as defined in Section 2.6. The text should read: Following the 1-week CBM phase (in the ITT sample), 19% of patients in the positive condition evidenced clinically significant change on the BDI-II compared to 21% in the control condition. Clinically significant change following iCBT was 44% in the positive condition and 26% in the control condition. The difference at both time points was not significant, *χ*^2^<3.5, *p*>.05.

